# Guardian of the Human Genome: Host Defense Mechanisms against LINE-1 Retrotransposition

**DOI:** 10.3389/fchem.2016.00028

**Published:** 2016-06-28

**Authors:** Yasuo Ariumi

**Affiliations:** Ariumi Project Laboratory, Center for AIDS Research and International Research Center for Medical Sciences, Kumamoto UniversityKumamoto, Japan

**Keywords:** LINE-1, retrotransposition, DNA double-strand breaks (DSBs), DNA repair, tumor suppressor, HBV, epigenetic regulation, somatic insertion

## Abstract

Long interspersed element type 1 (LINE-1, L1) is a mobile genetic element comprising about 17% of the human genome, encoding a newly identified ORF0 with unknown function, ORF1p with RNA-binding activity and ORF2p with endonuclease and reverse transcriptase activities required for L1 retrotransposition. L1 utilizes an endonuclease (EN) to insert L1 cDNA into target DNA, which induces DNA double-strand breaks (DSBs). The ataxia-telangiectasia mutated (ATM) is activated by DSBs and subsequently the ATM-signaling pathway plays a role in regulating L1 retrotransposition. In addition, the host DNA repair machinery such as non-homologous end-joining (NHEJ) repair pathway is also involved in L1 retrotransposition. On the other hand, L1 is an insertional mutagenic agent, which contributes to genetic change, genomic instability, and tumorigenesis. Indeed, high-throughput sequencing-based approaches identified numerous tumor-specific somatic L1 insertions in variety of cancers, such as colon cancer, breast cancer, and hepatocellular carcinoma (HCC). In fact, L1 retrotransposition seems to be a potential factor to reduce the tumor suppressive property in HCC. Furthermore, recent study demonstrated that a specific viral-human chimeric transcript, HBx-L1, contributes to hepatitis B virus (HBV)-associated HCC. In contrast, host cells have evolved several defense mechanisms protecting cells against retrotransposition including epigenetic regulation through DNA methylation and host defense factors, such as APOBEC3, MOV10, and SAMHD1, which restrict L1 mobility as a guardian of the human genome. In this review, I focus on somatic L1 insertions into the human genome in cancers and host defense mechanisms against deleterious L1 insertions.

## Introduction

Long interspersed element type 1 (LINE-1, L1) is an active and autonomous non-long terminal repeat (LTR) retrotransposon composing about 17% of the human genome and L1 is an essential evolutionary force (DeBerardinis et al., 1998; Ostertag and Kazazian, 2001; Cordaux and Batzer, 2009; Hancks and Kazazian, 2012). However, only 100 copies out of ~500,000 copies still remain active (Brouha et al., 2003; Mills et al., 2007; Beck et al., 2010). The remaining L1s are 5′ truncated and defective. Furthermore, L1 provides the *trans*-acting functions required for the retrotransposition of non-autonomous retrotransposons such as short interspersed element (SINE), which includes Alu repeats in humans, SINE-VNTR-Alu (SVA), and processed pseudogenes (Esnault et al., 2000; Dewannieux et al., 2003; Hancks et al., 2011).

L1 encodes three open reading frames, a newly identified ORF0 with unknown function, ORF1p with RNA-binding and nucleic acid chaperon activities, and ORF2p with AP-like endonuclease (EN) and reverse transcriptase (RT) activities required for L1 retrotransposition (Mathias et al., 1991; Martin and Bushman, 2001; Ostertag and Kazazian, 2001; Hancks and Kazazian, 2012; Denli et al., 2015). ORF0 is the primate-specific ORF in the anti-sense 5′ untranslated region (UTR) of L1 (Denli et al., 2015). ORF0 predominantly localizes in nuclear PML-adjacent foci and enhances L1 mobility. ORF1p and ORF2p preferentially assemble with L1 RNA and form a ribonucleoprotein (RNP) in the cytoplasmic foci (Goodier et al., 2007; Doucet et al., 2010). Although retroviruses and LTR-retrotransposons utilize a long terminal repeat (LTR) to synthesize full-length transcripts, L1 instead utilizes an internal promoter in the 5′UTR of L1 (Swergold, 1990). Several transcription factors including SOX11 (Tchenio et al., 2000), YY1 (Becker et al., 1993; Athanikar et al., 2004), RUNX3 (Yang et al., 2003), and p53 (Harris et al., 2009) positively regulate the L1 transcription. On the other hand, SOX2 (Muotri et al., 2005) and SRY (Tchenio et al., 2000) as well as several epigenetic factors negatively regulate the L1 transcription (Table 1).

**Table 1:** **Host factors regulating the L1 transcription**.

**Host factors**	**Functions**	**References**
**POSITIVE FACTOR**		
RNA polymerase II	RNA polymerase	Swergold, 1990; Beck et al., 2011
SOX11	Transcription factor	Tchenio et al., 2000
YY1	Transcription factor	Becker et al., 1993; Athanikar et al., 2004
RUNX3	Transcription factor	Yang et al., 2003
p53	Transcription factor	Harris et al., 2009
**NEGATIVE FACTOR**		
MeCP2	Methyl-CpG-binding protein	Yu et al., 2001; Muotri et al., 2010
KAP1/TRIM28	Cofactor of KRAB zinc finger protein	Rowe et al., 2010; Castro-Diaz et al., 2014
SETDB1/ESET	Histone methyltransferase	Matsui et al., 2010
DNMT1, DNMT3a, DNMT3b	DNA methyltransferase	Liang et al., 2002
ZNF93	KRAB zinc finger protein	Jacobs et al., 2014
PLZF	Transcription factor	Puszyk et al., 2013
SIRT6	Mono-ADP-ribosyl transferase	Van Meter et al., 2014
SOX2	Transcription factor	Muotri et al., 2005
SRY	Transcription factor	Tchenio et al., 2000
p53	Tumor suppressor	Wylie et al., 2016
Rb	Tumor suppressor	Montoya-Durango et al., 2009, 2016
HDAC1	Histone deacetylase	Montoya-Durango et al., 2009
HDAC2	Histone deacetylase	Montoya-Durango et al., 2009, 2016
E2F	Transcription factor	Montoya-Durango et al., 2009
NuRD	Nucleosomal and remodeling deacetylase	Montoya-Durango et al., 2016

**Figure 1:**
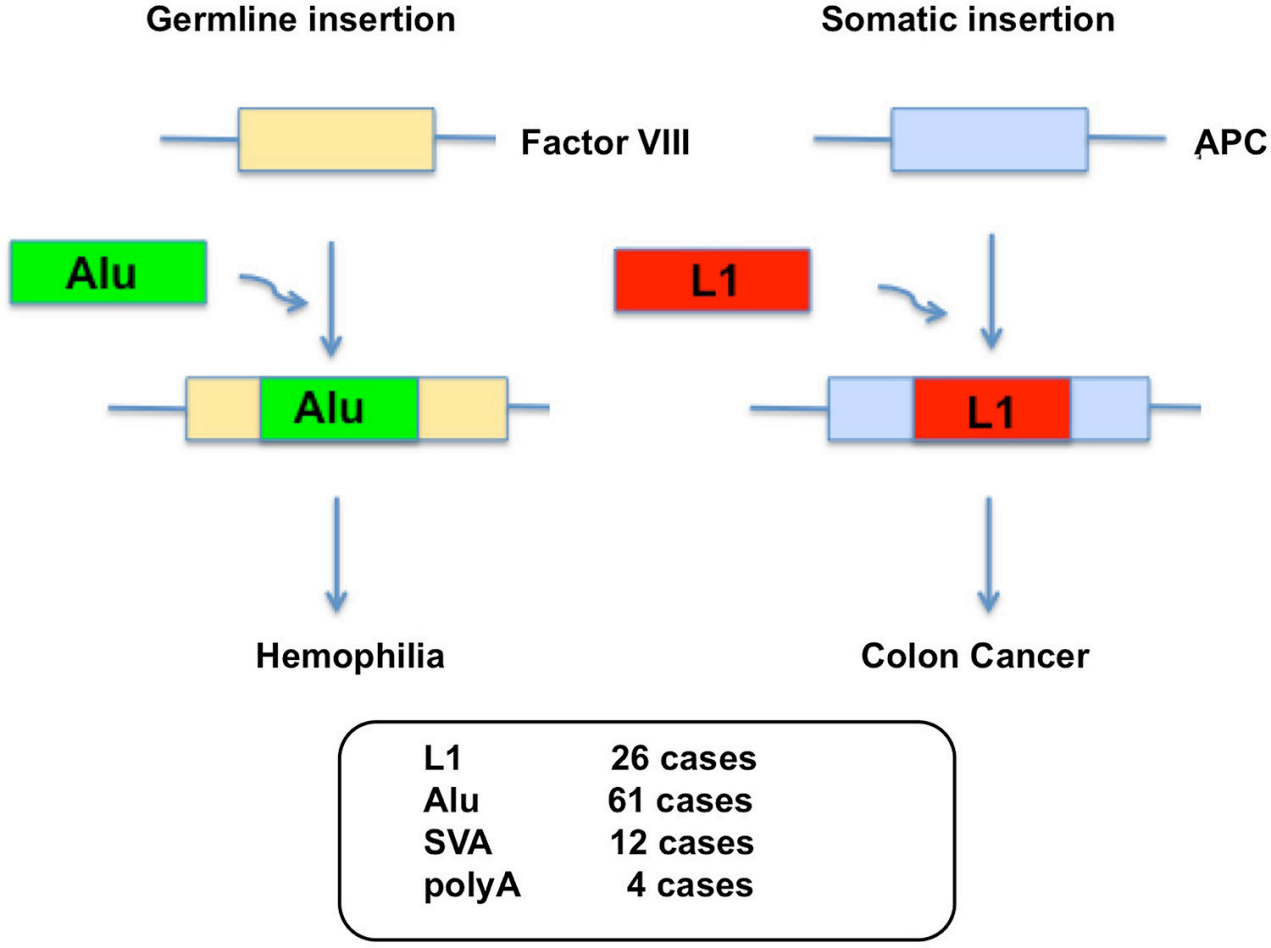
**Germline Alu insertion in the human genome causes hemophilia and somatic L1 insertion causes colon cancer**. L1 insertions have also been observed in Factor VIII and this causes hemophilia A. Disease causing L1 and Alu insertions are also found in genes that are X-linked inherited. >100 disease-causing retrotransposon insertions have been identified in humans [26 L1, 61 Alu, 12 SVA, 4 poly(A)].

L1 integrates into the genome by target-primed reverse transcription (TPRT) (Luan et al., 1993) after the L1-RNP complex enters the nucleus. During TPRT, the L1 EN creates a nicked DNA that serves as a primer for reverse transcription of L1 RNA, leading to integration of L1 cDNA into the human genome (Feng et al., 1996). A typical L1 EN cleavage site is 5′-TTTT/AA-3′ (Feng et al., 1996; Cost and Boeke, 1998). Thus, L1 insertion generates DNA double-strand breaks (DSBs) as well as L1 structural hallmarks such as frequent 5′ truncations, 3′ poly(A) tails and variable length target site duplications (TSDs) in the target DNA. L1 can alter the mammalian genome in many ways upon retrotransposition, since the insertion of L1 into the human genome may cause genomic instability, genetic disorders, and cancers through insertional mutagenesis (Kazazian et al., 1988; Morse et al., 1988; Miki et al., 1992; Narita et al., 1993; Holmes et al., 1994; Gilbert et al., 2002; Morrish et al., 2002; Symer et al., 2002; Belancio et al., 2008; Beck et al., 2011; Hancks and Kazazian, 2012; Bundo et al., 2014; Kines et al., 2014; Figure 1). So far, >100 disease-causing retrotransposon insertions have been identified in humans [26 L1, 61 Alu, 12 SVA, 4 poly(A)] (Figure 1).

## L1-mediated DSBs induction and DNA repair machinery

L1 is known to induce DSBs in target DNA by L1 EN activity (Gasior et al., 2006). The ataxia-telangiectasia mutated (ATM) is activated by DSBs and subsequently phosphorylates downstream substrates including p53, Chk2, BRCA1 and the MRE11-Rad50-NBS1 (MRN) complex, resulting in the activation of DNA damage checkpoint and cell cycle arrest (Harper and Elledge, 2007; Ciccia and Elledge, 2010; Shiloh, 2014). Accordingly, L1 retrotransposition was increased in ATM-deficient cells, indicating ATM signaling pathway modulates L1 retrotransposition (Coufal et al., 2011). In contrast, the E6 protein from β-human papillomavirus (β-HPV 5 and 8) reduces ATM protein levels and attenuates L1 retrotransposition, suggesting that ATM is needed for efficient L1 retrotransposition (Wallace et al., 2013). Thus, the DNA damage response may modulate L1 retrotransposition. Notably, L1 can integrate into preformed DSBs generated independently of L1 EN, resulting in retrotransposon-mediated DNA repair (Morrish et al., 2002). Furthermore, host DNA repair machinery may also impact L1 retrotransposition. Gasior et al reported that DNA repair enzyme ERCC1/XPF heterodimer limits L1 retrotransposition (Gasior et al., 2008). Importantly, deficiencies of non-homologous end-joining (NHEJ) repair pathway such as Ku70, Artemis, and DNA ligase IV (LigIV) decrease retrotransposition frequencies of human L1 in chicken DT40 cells, suggesting that the NHEJ repair pathway is required for efficient L1 retrotransposition (Suzuki et al., 2009).

## L1 retrotransposition in cancers

Somatic L1 insertions are seldom observed in normal tissues except hippocampus (Baillie et al., 2011; Evrony et al., 2012; Upton et al., 2015). Although most L1 retrotransposition was thought to occur in the germline, somatic L1 insertions were also found to occur in variety of tumors, including breast cancer, colon cancer, hepatocellular carcinoma (HCC), and lung cancer (Miki et al., 1992; Liu et al., 1997; Iskow et al., 2010; Lee et al., 2012; Solyom et al., 2012; Shukla et al., 2013; Carreira et al., 2014; Helman et al., 2014; Ewing et al., 2015; Table 2). First, three L1 insertion candidates were reported in human tumors (Morse et al., 1988; Miki et al., 1992; Liu et al., 1997). However, two insertions described by Liu et al. (1997) and Morse et al. (1988) lack all of the hallmark features of a true somatic retrotransposition event, such as L1 endonuclease cleavage site, the presence of L1 poly(A) tail, target-site duplication (TSD), 5′ truncation and inversion, and 3′ transduction (Holmes et al., 1994; Moran et al., 1999; Goodier et al., 2000; Pickeral et al., 2000; Szak et al., 2002; Table 2). These insertions may be derived from recombination events, L1 EN-independent insertions (Morrish et al., 2002), or other atypical integration mechanisms of L1 retrotransposition. Indeed, an L1 insertion disrupts the adenomatous polyposis coli (APC) gene in a colon cancer, indicating the disruption of a tumor suppressor gene caused by somatic L1 insertion (Miki et al., 1992). Accordingly, a recent study identified a novel somatic insertion in the APC gene and a hot spot for L1 insertion on Chromosome 17, suggesting that the L1 insertion initiates colorectal cancer (CRC) by mutating the APC gene through the classic two-hit CRC pathway (Scott et al., 2016). Furthermore, high-throughput sequencing-based approaches identified numerous somatic tumor-specific insertions in cancers (Miki et al., 1992; Liu et al., 1997; Iskow et al., 2010; Lee et al., 2012; Solyom et al., 2012; Shukla et al., 2013; Carreira et al., 2014; Helman et al., 2014; Tubio et al., 2014). Indeed, Lee et al. identified the L1 insertions in cadherin-12 (CDH12), roundabout, axon guidance receptor, homolog 2 (ROBO2), NRXN3, FPR2, COL11A1, NEGR1, NTM, and CTNNA2 (Lee et al., 2012). As well, Solyom et al. identified several tumor-specific insertions in colorectal tumors including odd Oz/ten-m homolog 3 (ODZ3), ROBO2, protein tyrosine phosphatase, receptor type, M (PTPRM), pericentriolar material 1 (PCM1), CDH11, and runt-related transcription factor 1 (RUNX1T1) of colorectal cancers (Solyom et al., 2012). All insertions were severely 5′ truncated. Interestingly, these genes are associated with cell-adhesion functions and both groups could identify the L1 insertions in the same ROBO2 genes, suggesting the potential role of cell-adhesion genes in L1 insertion-mediated colorectal tumorigenesis. In addition, Tubio et al. analyzed the somatic L1 retrotransposition activities in 290 cancers and noticed insertions occurring during cancer development. 53% of the patients have at least one somatic L1 retrotransposition event, of which 24% were 3′ transductions, most frequently colorectal cancers (93%) and lung cancers (75%), suggesting that 3′ transduction are potentially mutagenic. Somatic L1 retrotranspositions tend to insert in intergenic or heterochromatin regions of the cancer genome (Tubio et al., 2014). Furthermore, somatic L1 insertions participate in the dynamics of many tumor genomes and lead to driver mutations. Surprisingly, L1 insertion was reported in colonic adenoma, a known cancer precursor, suggesting that widespread somatic L1 retrotransposition occurs early during development of gastrointestinal (GI) tumors, probably before dysplastic growth (Ewing et al., 2015). Similarly, a recent study demonstrated that L1 retrotransposition is active in esophageal adenocarcinoma and its precursor, Barrett's esophagus (BE), indicating that somatic L1 insertions occur early in BE and esophageal adenocarcinoma. Notably, two L1 insertions were detected in normal esophagus, indicating that some L1 insertions may occur in normal squamous epithelium cells (Doucet-O'Hare et al., 2015). In this regard, most of the new somatic insertions are truncated, and would not mobilize again. So mutations arising from insertions in the normal precursor esophageal or benign BE would be contributing to tumorigenesis. Otherwise, only a rare full-length somatic insertion has the potential to contribute to mutation during the various stages of transition to tumorigenesis. In addition, L1 insertions in pancreatic ductal adenocarcinoma (PDAC) were reported with discordant rate of retrotransposition between primary and metastatic sites, suggesting that L1 insertions in gastrointestinal neoplasms occur discontinuously. Thus, somatic L1 insertions contribute to genetic and phenotypic heterogeneity in PDAC (Rodić et al., 2015). Interestingly, somatic insertions were identified in epithelial tumors but not in blood or brain cancers (Lee et al., 2012). However, we raise awareness regarding the following limitations of this study. For example, the sample size was small and the normal tissue was not from the same patient. In addition, in this study they only examined multiple myeloma and did not look at the entire spectrum of blood based cancers. In this regard, ten-eleven-translocation (TET) 2, a DNA demethylation-related protein, is frequently mutated in myeloid and lymphoid tumors (Ko et al., 2015). The TET family that oxidizes 5-methylcytosine (5mC) to 5-hydroxymethylcytosine (5hmC), 5-formylcytosine (5fC), and 5-carboxylcytosine (5caC) in DNA, leads to the DNA demethylation. Since DNA methylation has a pivotal regulatory role in L1 silencing, TET2 may impact L1 mobility. Therefore, L1 insertions may be suppressed in such hematological cancers. Intriguingly, several somatic insertions occur in genes that are commonly mutated in cancers such as tumor suppressor gene. These insertions disrupt the expression of target genes, and are biased toward regions of cancer-specific DNA hypomethylation (Lee et al., 2012). Indeed, recent studies identified somatic L1 insertion in tumor suppressor genes, such as APC and PTEN (Miki et al., 1992; Helman et al., 2014). As well, the first case of familial retinoblastoma (Rb) caused by a *de novo* insertion of a full-length L1 into intron 14 of the Rb gene, resulting in the aberrant and non-canonical mRNA splicing of the Rb gene, was reported (Rodríguez-Martín et al., 2016). Furthermore, 18 retrotransposon insertions [14 Alu, 3 L1, and 1 poly(A)] were identified in neurofibromatosis type 1 (NF1) gene (Wimmer et al., 2011).

**Table 2:** **L1 insertions in cancers**.

**Tumor type**	**L1 insertions and target genes**	**References**
Barrett's esophagus (BE)	+ 46 somatic L1 insertions	Doucet-O'Hare et al., 2015
Breast cancer	+ Myc (^*^)	Morse et al., 1988
Colorectal cancer	+ APC	Miki et al., 1992; Scott et al., 2016
	+ ODZ3, ROBO2, PTPRM, PCM1, CDH11, RUNX1T1	Solyom et al., 2012
	+ 25 somatic L1 insertions ROBO2, CDH12, NRXN3, FPR2 COL11A1, NEGR1, NTM, CTNNA2	Lee et al., 2012
	+ 57 somatic L1 insertions CYLD, HDAC9	Ewing et al., 2015
Colonic adenoma	+ 29 somatic L1 insertions STX11, PANX1	Ewing et al., 2015
Desmoplastic small round cell tumor	+ t(11;22) translocation breakpoint(^*^) EWS-WT1	Liu et al., 1997
Endometrial carcinoma	+ PTEN	Helman et al., 2014
Esophageal adenocarcinoma	+ 75 somatic L1 insertions	Doucet-O'Hare et al., 2015
Familial retinoblastoma	+ RB1	Rodríguez-Martín et al., 2016
Gastric cancer	+ 23 somatic L1 insertions ELOVL4, CNTNAP2, RIMS2	Ewing et al., 2015
Glioblastoma	−	Iskow et al., 2010; Lee et al., 2012
Hepatocellular carcinoma (HCC)	+ MCC, ST18	Shukla et al., 2013
	HBV integration in L1 (HBx-LINE1)	Lau et al., 2014
Head and neck carcinoma	+	Helman et al., 2014
Lung cancer	+ 9 somatic L1 insertions	Iskow et al., 2010
Medulloblastoma	−	Iskow et al., 2010
Multiple Myeloma	−	Lee et al., 2012
Neurofibromatosis type 1 (NF1)	+ NF1 3 L1 insertions	Wimmer et al., 2011
Ovarian tumors	+ 13 somatic L1 insertions	Lee et al., 2012
Pancreatic cancer	+ 24 somatic L1 insertions SOX6, APAF1, GDNF	Ewing et al., 2015
Pancreatic ductal	+ 465 somatic L1 insertions	Rodić et al., 2015
adenocarcinoma (PDAC)	In 20 PDAC cases	
Prostate tumors	+	Lee et al., 2012

* *Lack of the hallmark features of a true somatic retrotransposition event (Morse et al., 1988; Liu et al., 1997)*.

Although still debated, cell division seems to be required for efficient L1 retrotransposition (Shi et al., 2007; Xie et al., 2013). In fact, retrotransposition was strongly inhibited in the cells arrested in the G_1_, S, G_2_, or M phase of cell cycle. The reduction in L1 transcript abundance limits retrotransposition in non-dividing cells, suggesting that inhibition of retrotransposition in non-dividing cells protects somatic cells from accumulation of deleterious mutations caused by L1 insertions (Shi et al., 2007). In contrast, there is an opposite report that L1 retrotransposition was detected in non-dividing and primary human somatic cells using adenovirus-L1 hybrid vector, even though they detected L1 retrotransposition in G_1_/S- but not in G_0*-*_arrested cells (Kubo et al., 2006). In addition, retrotransposition was also inhibited during cellular senescence in primary human fibroblasts. So far, several biomarkers of cellular senescence have been identified such as senescence-associated β-galactosidase (SA-β-Gal), p53/p21, p16^INK4a^, senescence-associated heterochromatin foci (SAHF), senescence-associated secretory phenotype (SASP), autophagy, telomere-induced foci/DNA damage response (DDR), and cell cycle arrest (Kuilman et al., 2010) and the reduction in L1 retrotransposition may be a biomarker of cellular senescence. Thus, cell cycle may affect L1 retrotransposition.

L1 protein expression is a common feature of many types of high-grade malignant tumor, yet is rarely detected in early stage of tumorigenesis (Rodić et al., 2014). L1 promoter is normally silenced by methylation in normal somatic cells (Woodcock et al., 1997; Schulz et al., 2006). In contrast, L1 promoter is hypomethylated (Baba et al., 2014), and expression of L1 is elevated in many tumors. In fact, L1 expression was detected in human breast carcinomas and testicular cancers (Bratthauer and Fanning, 1992; Bratthauer et al., 1994; Nangia-Makker et al., 1998). L1 ORF1p protein is detected in a variety of tumor cells including breast cancer, colon cancer, pancreatic ductal adenocarcinoma, and HCC but not in normal somatic cells (Bratthauer et al., 1994; Asch et al., 1996; Rodić et al., 2014). Thus, L1 ORF1p expression seems to be a hallmark of many human cancers as a highly specific tumor marker.

In addition to expression of L1, a hallmark of tumor cells is an activated telomere maintenance mechanism that allows prolonged survival of the malignant tumor cells. In more than 80% of tumors, telomeres are typically maintained by telomerase. Notably, the reduced length of telomeres was reported in the L1 knockdown cells, indicating that L1 is involved in telomere maintenance in telomerase positive tumor cells (Aschacher et al., 2016). Accordingly, L1 involves in a transcriptional regulation of hTERT and upregulation of its transcription factors c-Myc and KLF-4 (Aschacher et al., 2016). Thus, L1 may contribute to the development of cancers. However, these studies were not done in alternative lengthening of telomeres (ALT)-positive tumors or telomerase negative tumors. Consequently, it is uncertain if L1 is directly contributing to telomere maintenance or if the reduction in telomere length is contributed to the reduction in telomerase levels. Indeed, the stoichiometry of telomerase is important for maintaining telomere length (Armanios et al., 2005; Goldman et al., 2005).

**Figure 2:**
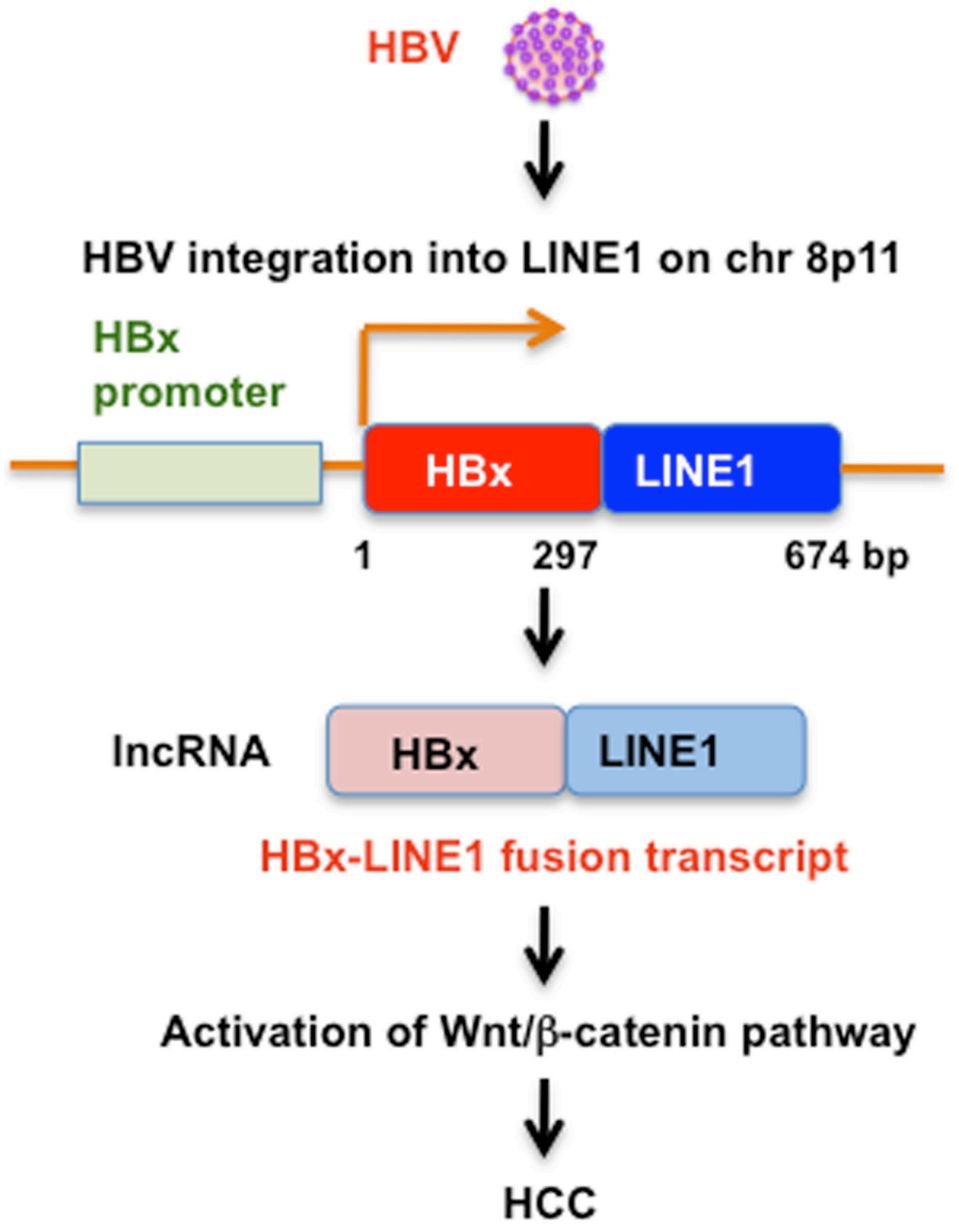
**HBV integration into LINE1 in HCC**. Schematic representation of the HBx-LINE1 fusion RNA transcript and potential role of oncogenic HBx-LINE1 fusion RNA transcript in HCC.

Chronic infection with hepatitis B virus (HBV) is a major risk for the development of HCC. HBV integration into the human genome was found in most HBV-related HCC and it has been implicated in the development of HCC. An initial study proposed that HBV integration occurs randomly without preferred integration site (Matsubara and Tokino, 1990). However, high-throughput sequencing-based approaches identified recurrent integration sites in HCC (Ding et al., 2012). HBV integration favored chromosome 17 and preferentially integrated into human transcript units. At least, telomerase reverse transcriptase (TERT) and fibronectin 1 (FN1) genes were identified as the recurrent HBV integration sites. Furthermore, seven integrations were found in the repeat regions including L1, LTR/ERV1, and SINE/Alu (Ding et al., 2012). Similarly, a recent transcriptome sequencing study of HBV-positive HCC cell lines discovered that HBV integrates into L1 (Lau et al., 2014). Insertion of the gene encoding hepatitis B virus x (HBx) into L1 on chromosome 8p11 produces an oncogenic HBx-LINE1 chimeric RNA transcript (Lau et al., 2014; Figure 2). The HBx-LINE1 RNA transcript was detected in 23.3% of HCC, suggesting that HBx-LINE1 is selected for in HCC oncogenesis. The long non-coding RNA (lncRNA)-like HBx-LINE1 transcript confers cancer-promoting properties through activation of Wnt/β-catenin signaling pathway (Lau et al., 2014).

Furthermore, endogenous L1-mediated retrotransposition was identified in the germline and somatic cells of HCC patients (Shukla et al., 2013). The germline L1 insertion in the tumor suppressor mutated in colorectal cancers (MCC) was detected in 21.1% of HCC, resulting in the aberrant expression of MCC. Moreover, suppression of tumorigenicity 18 (ST18) was activated by a tumor-specific somatic L1 insertion (Shukla et al., 2013). Thus, L1-mediated retrotransposition seems to be a potential etiological factor in HCC.

## Guardian of the human genome: Host defense mechanisms against L1 retrotransposition

Since insertion of L1 into the human genome may cause human genetic disorders and cancer, retrotransposition must be silenced under normal conditions. To restrict deleterious retrotransposition, host cells have evolved several defense mechanisms protecting cells against retrotransposition including epigenetic regulation through DNA methylation (Burden et al., 2005; Trono, 2015), RNA silencing by RNA interference (Soifer et al., 2005; Yang and Kazazian, 2006), PIWI-interacting RNA (piRNA)-PIWI system (Aravin et al., 2007a,b; Kuramochi-Miyagawa et al., 2008; De Fazio et al., 2011; Marchetto et al., 2013), microRNA (Hamdorf et al., 2015), and host restriction factors, such as apolipoprotein B mRNA editing enzyme catalytic polypeptide-like 3 (APOBEC3), Moloney leukemia virus 10 (MOV10), and SAM domain and HD domain containing protein 1 (SAMHD1) (Table 3).

**Table 3:** **Host defense factors against L1**.

**Host factors**	**Functions**	**References**
APOBEC3A	ISG, cytidine deaminase	Bogerd et al., 2006; Chen et al., 2006; Muckenfuss et al., 2006; Kinomoto et al., 2007; Niewiadomska et al., 2007
APOBEC3B	Cytidine deaminase	Bogerd et al., 2006; Muckenfuss et al., 2006; Stenglein and Harris, 2006; Kinomoto et al., 2007
APOBEC3F	ISG, cytidine deaminase anti-viral protein	Muckenfuss et al., 2006; Stenglein and Harris, 2006; Kinomoto et al., 2007; Niewiadomska et al., 2007
APOBEC3G	ISG, cytidine deaminase anti-viral protein	Kinomoto et al., 2007; Niewiadomska et al., 2007
MOV10	ISG, RNA helicase anti-HIV protein	Arjan-Odedra et al., 2012; Goodier et al., 2012, 2013; Li et al., 2013
BST-2	ISG, anti-HIV protein	Goodier et al., 2015
ISG20	ISG, anti-viral protein	Goodier et al., 2015
MAVS	ISG, innate immunity	Goodier et al., 2015
Mx2	ISG, anti-viral protein	Goodier et al., 2015
RNase L	ISG, anti-viral protein	Zhang et al., 2014
SAMHD1	ISG, anti-HIV protein	Zhao et al., 2013; Hu et al., 2015
TREX1	ISG, DNA exonuclease	Stetson et al., 2008
ZAP/PARP13	ISG, poly(ADP-ribose) polymerase	Goodier et al., 2015; Moldovan and Moran, 2015
IFN1	Induction of ISGs, anti-viral protein	Yu et al., 2015
MeCP2	Methyl-CpG-binding protein	Yu et al., 2001; Muotri et al., 2010
KAP1/TRIM28	Cofactor of KRAB zinc finger protein	Rowe et al., 2010; Castro-Diaz et al., 2014
SETDB1/ESET	Histone methyltransferase	Matsui et al., 2010
ZNF93	KRAB zinc finger protein	Jacobs et al., 2014
PLZF	Transcription factor	Puszyk et al., 2013
SIRT6	Mono-ADP-ribosyl transferase	Van Meter et al., 2014
SOX2	Transcription factor	Muotri et al., 2005
SRY	Transcription factor	Tchenio et al., 2000
p53	Tumor suppressor	Wylie et al., 2016
Rb	Tumor suppressor	Montoya-Durango et al., 2009, 2016
ATM	DNA damage sensor, kinase	Coufal et al., 2011
ERCC1/XPF	DNA repair	Gasior et al., 2008
miR-128	microRNA	Hamdorf et al., 2015
piRNA-PIWI	piRNA	De Fazio et al., 2011; Marchetto et al., 2013

**Figure 3:**
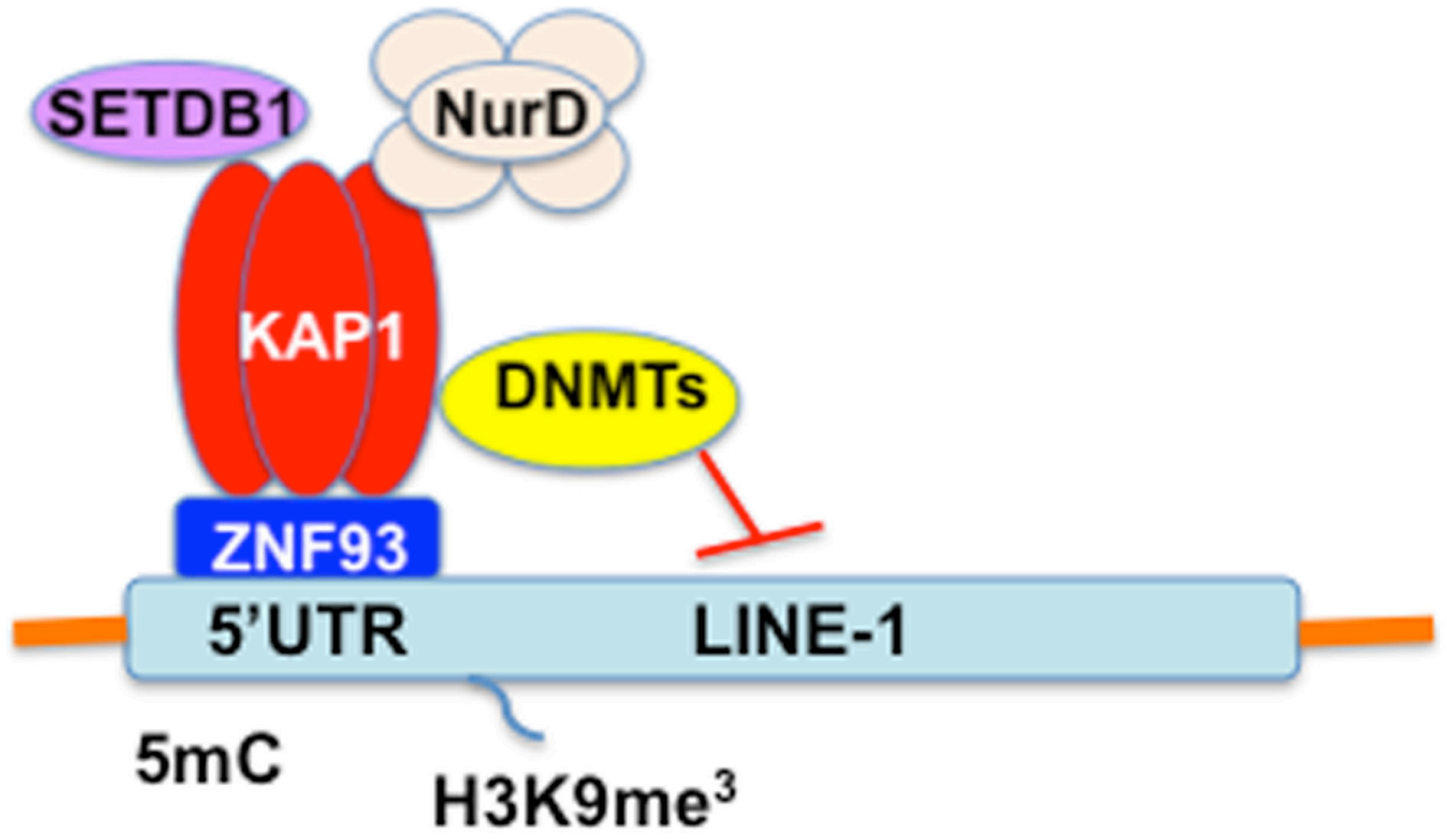
**The KRAB/KAP1 system controls the transcriptional activity of L1 in ES cells**. KRAB/KAP1 serves as a scaffold for a heterochromatin complex comprising the SETDB1 and DNMT.

DNA methylation within the 5′UTR promoter of L1 is essential for maintaining transcriptional inactivation and for inhibiting L1 retrotransposition (Woodcock et al., 1997; Liang et al., 2002; Burden et al., 2005). L1 is highly active during early embryogenesis, while L1 is silenced early in development through epigenetic mechanisms (Table 1). Indeed, methylation of the L1 promoter is maintained by DNA metyltransferases (DNMTs), including DNMT1, DNMT3a, and DNMT3b (Liang et al., 2002). L1 retrotransposition is negatively regulated by methyl-CpG-binding protein 2 (MeCP2)-mediated DNA methylation (Yu et al., 2001; Muotri et al., 2010). In addition, nucleosomal and remodeling deacetylase (NuRD) multiprotein complex specifically enriches the L1 promoter. Rb and E2F recruit to the L1 promoter along with histone deacetylase (HDAC), including HDAC1 and HDAC2 (Montoya-Durango et al., 2009, 2016). Furthermore, KRAB-associated protein1 (KAP1, also known as TRIM28) mediates transcriptional silencing of endogenous retroelements (EREs) including L1, Alu, SVA, and human endogenous retrovirus-K (HERV-K) as well as exogenous retrovirus mouse leukemia virus (MLV) in embryonic stem (ES) cells (Wolf and Goff, 2007, 2009; Matsui et al., 2010; Rowe et al., 2010; Castro-Diaz et al., 2014; Turelli et al., 2014; Trono, 2015). Krüppel-associated box (KRAB)-containing zinc-finger protein (KRAB-ZFP/ZNF), a large family of tetrapod-restricted transcription factors, and a cofactor KAP1 serve as a scaffold for a heterochromatin complex comprising the SETDB1 (also known as ESET) histone methyltransferase, histone deacetylase, nucleosome remodeling, and DNMT activities (Trono, 2015; Figure 3). Furthermore, the protein deacylase and mono-ADP ribosyltransferase Sirtuin 6 (SIRT6) represses L1 mobility by ribosylating KAP1 (Van Meter et al., 2014). SIRT6 binds to the 5′UTR of L1 and ribosylates KAP1, resulting in facilitation of KAP1 interaction with the heterochromatin factor HP1α, thereby contributing to the packaging of L1 into transcriptionally repressive heterochromatin. Moreover, promyelocytic leukemia zinc finger (PLZF) protein, a member of the POK (POZ and Kruppel zinc finger) family of transcription factors that acts as an epigenetic regulator of stem cell maintenance in germ cells and haematopoietic stem cells, represses L1 retrotransposition in germ and progenitor cells (Puszyk et al., 2013). PLZF-mediated DNA methylation induces silencing of the L1 gene, resulting in inhibition of L1 retrotransposition. Species-specific KZNFs might recruit KAP1 to species-specific retrotransposon classes that recently invaded the host genome. In this regard, Jacobs et al. recently reported that two primate-specific ZNF91 and ZNF93 repress SVA and L1 retrotransposons, respectively (Jacobs et al., 2014). ZNF93 evolved earlier to repress the primate L1 lineage until ~12.5 million years ago when the L1PA3 subfamily escaped ZNF93-mediated restriction through the removal of the ZNF93-binding site, suggesting an evolutionary arms race between KRAB-ZNFs and retrotransposons (Jacobs et al., 2014).

Post-translational modification and subcellular localization of L1 protein seem to be important for modulation of L1 mobility. In fact, phosphorylation of ORF1p is required for L1 retrotransposition (Cook et al., 2015). L1 ORF1p contains four conserved proline-directed protein kinase (PDPKs) target sites. PDPK mutations in ORF1p could inactivate L1 mobility (Cook et al., 2015). The PDPK family includes mitogen-activated protein kinases (MAPKs) and cyclin-dependent kinases (CDKs). Although nuclear localization of L1 ORF1p and ORF2p is essential for L1 retrotransposition, L1 ORF1p predominantly localizes in punctate cytoplasmic foci in most of cases (Goodier et al., 2007; Harris et al., 2010; Chen et al., 2012). However, in several breast cancers, LI ORF1p and ORF2p were also detected in the nucleus (Harris et al., 2010; Chen et al., 2012). Indeed, the expression of L1 is elevated in breast cancers.

Recently, APOBEC3 family of cytidine deaminases, MOV10, and SAMHD1 have been identified as restriction factors for human immunodeficiency virus type 1 (HIV-1) (Sheehy et al., 2002; Harris et al., 2003; Mangeat et al., 2003; Zhang et al., 2003; Burdick et al., 2010; Furtak et al., 2010; Wang et al., 2010; Hrecka et al., 2011; Laguette et al., 2011). APOBEC3A, APOBEC3B, and APOBEC3F but not APOBEC3G inhibit L1 retrotransposition by a DNA deaminase-independent manner, indicating a novel anti-L1 retrotransposition mechanism (Turelli et al., 2004; Bogerd et al., 2006; Chen et al., 2006; Muckenfuss et al., 2006; Stenglein and Harris, 2006; Hulme et al., 2007; Kinomoto et al., 2007; Niewiadomska et al., 2007; Schumann, 2007; Arias et al., 2012). In contrast, Kinomoto et al. and Niewiadomska et al. reported that APOBEC3G could inhibit L1 retrotransposition by a DNA deamination-independent manner (Kinomoto et al., 2007; Niewiadomska et al., 2007). Furthermore, APOBEC3G inhibits Alu retrotransposition by a DNA deaminase-independent manner (Chiu et al., 2006; Hulme et al., 2007; Bulliard et al., 2009). MOV10 RNA helicase also inhibits L1 and Alu retrotransposition (Arjan-Odedra et al., 2012; Goodier et al., 2012, 2013; Li et al., 2013). Similarly, SAMHD1 inhibits LINE-1 and Alu/SVA retrotransposition (Zhao et al., 2013). SAMHD1 inhibits L1 retrotransposition through promoting the sequestration of L1 RNP within stress granules (Hu et al., 2015). Similarly, the zinc-finger antiviral protein (ZAP) also known as PARP13, a member of poly(ADP-ribose) polymerase (PARP) family, inhibits the retrotransposition of L1, Alu, and intracisternal A particle (IAP) retrotransposons (Goodier et al., 2015; Moldovan and Moran, 2015). ZAP interacts with L1 RNA and L1 ORF1p and co-localizes with stress granules.

Type I interferons (IFN 1) including IFNα and IFNβ have been involved in innate immunity against viruses. In this regard, a recent study reported that L1 induces IFN1 and IFN1, in turn, inhibits L1 retrotransposition, suggesting that IFN1 controls propagation of L1 as well as maintenance of genomic integrity (Yu et al., 2015). Accordingly, several interferon-stimulated genes (ISGs), including APOBEC3, MOV10, BST-2, ISG20, MAVS, MX2, RNase L, SAMHD1, TREX1, and ZAP restrict L1 retrotransposition, indicating that ISGs are key players of the type I interferon anti-retroelement response (Turelli et al., 2004; Bogerd et al., 2006; Chen et al., 2006; Muckenfuss et al., 2006; Stenglein and Harris, 2006; Hulme et al., 2007; Kinomoto et al., 2007; Niewiadomska et al., 2007; Schumann, 2007; Stetson et al., 2008; Arias et al., 2012; Zhao et al., 2013; Zhang et al., 2014; Goodier et al., 2015; Hu et al., 2015; Table 3).

Small RNAs have been implicated in the regulation of L1 mobility. Piwi proteins and Piwi-interacting RNAs (piRNA) silence L1 during genome reprogramming in the embryonic male germ line (De Fazio et al., 2011; Marchetto et al., 2013). Notably, Hamdorf et al. uncovered a new mechanism in which microRNAs restrict L1 mobilization and L1-associated mutations in cancer cells, cancer-initiating cells and iPS cells (Hamdorf et al., 2015). Indeed, miR-128 represses L1 retrotransposition by binding directly to L1 RNA, suggesting a new function of microRNAs in mediating genomic stability by suppressing the mobility of endogenous retrotransposons.

Tumor suppressor p53 mutations occur in most of human cancers, however, precisely how p53 functions to mediate tumor suppression is not well understood. In this regard, p53 was reported to restrict L1 mobility and suggested that p53 restricts oncogenesis in part by restricting transposon mobility (Wylie et al., 2016). Although normal human p53 suppressed transposons, mutant p53 from cancer patients could not. In contrast, L1 activity was elevated in p53 negative human cancers. Thus, ancestral function of p53 may be associated with transposon control as a guardian of human genome.

## Conclusion

L1 has successfully propagated and composed 17% of the human genome, resulting in evolutionary force. Activation of the normally silent L1 is associated with a high level of cancer-associated DNA damage and genomic instability. Indeed, L1 insertions into the human genome may cause cancers through insertional mutagenesis. In fact, recent high-throughput sequencing-based approaches could identify numerous somatic tumor-specific L1 insertions in a variety of cancers (Iskow et al., 2010; Lee et al., 2012; Solyom et al., 2012; Shukla et al., 2013; Helman et al., 2014), however there is no sufficient evidence. Therefore, it should clarify the role of L1-mediated retrotransposition in human cancers. Indeed, the implication of L1 insertion events as either passenger or driver mutations with a causative role in tumorigenesis still remains to be clarified (Rodić and Burns, 2013). Intriguingly, somatic insertions were only identified in epithelial tumors (Lee et al., 2012). Accordingly, epithelial cells can be transformed to cancer stem cells (Wang et al., 2013) and metastasis is more prevalent in epithelial tumors (Gotzmann et al., 2004). Thus, epithelial cells seem to be plastic (Carreira et al., 2014). Cancer stem cells are defined as rare cells with indefinite potential for self-renewal that drive tumorigenesis (Reya et al., 2001). However, it remains to be clarified the role of L1 mobility in cancer stem cells. Recent studies focus on the relationship among L1 mobility, reprogramming, and differentiation. Indeed, reprogramming somatic cells into iPS cells activates L1 mobility (Wissing et al., 2012; Friedli et al., 2014; Klawitter et al., 2016). On the other hand, L1 mobility is enhanced in tumor cells. In this regard, the elevation of L1 protein or RNA expression levels may be useful as a diagnostic hallmark of many human cancers and as a tumor specific marker, metastasis, and prognosis. Furthermore, recent advances in single cell analysis will be useful for comparison of the L1 mobility and the integration site of L1 at a single cell level in human cancers.

Finally, tumor suppressor proteins may be associated with transposon control to restrict deleterious retrotransposition as a guardian of the human genome. Wild-type p53 suppresses transposon mobility in normal cells, while mutant p53 in cancer cells could not, resulting in the activation of L1 mobility in cancer cells (Wylie et al., 2016). Furthermore, recent studies identified somatic L1 insertion in tumor suppressor genes, such as APC, PTEN, NF1, and Rb (Miki et al., 1992; Wimmer et al., 2011; Helman et al., 2014; Rodríguez-Martín et al., 2016). Thus, L1 insertions in the tumor suppressor genes may disrupt their functions and be associated with tumorigenesis. Altogether, host cells have evolved several defense mechanisms protecting cells against retrotransposition.

## Author contributions

The author confirms being the sole contributor of this work and approved it for publication.

## Funding

This work was supported by a Lateral Research from the Japan Society for the Promotion of Science (JSPS), by the Research Program on Hepatitis from Japan Agency for Medical Research and Development, AMED, and by Takeda Science Foundation.

### Conflict of interest statement

The author declares that the research was conducted in the absence of any commercial or financial relationships that could be construed as a potential conflict of interest.
